# Community assembly of gut microbiomes in yolk sac fry of Atlantic salmon: host genetics, environmental microbiomes, and ecological processes

**DOI:** 10.1093/femsec/fiaf007

**Published:** 2025-01-17

**Authors:** Amalie Johanne Horn Mathisen, Sol Gómez de la Torre Canny, Madeleine S Gundersen, Mari-Ann Østensen, Yngvar Olsen, Olav Vadstein, Ingrid Bakke

**Affiliations:** Department of Biotechnology and Food Science, NTNU Norwegian University of Science and Technology, 7491 Trondheim, Norway; Department of Biotechnology and Food Science, NTNU Norwegian University of Science and Technology, 7491 Trondheim, Norway; Skretting Aquaculture Innovation (AI), 4016 Stavanger, Norway; Department of Biotechnology and Food Science, NTNU Norwegian University of Science and Technology, 7491 Trondheim, Norway; Department of Biology, NTNU Norwegian University of Science and Technology, 7491 Trondheim, Norway; Department of Biology, NTNU Norwegian University of Science and Technology, 7491 Trondheim, Norway; Department of Biotechnology and Food Science, NTNU Norwegian University of Science and Technology, 7491 Trondheim, Norway; Department of Biotechnology and Food Science, NTNU Norwegian University of Science and Technology, 7491 Trondheim, Norway

**Keywords:** yolk sac stage, Atlantic salmon, gut microbiomes, community assembly, host genetics

## Abstract

In this study, we investigated the influence of host genetics and environmental microbiomes on the early gut microbiome of Atlantic salmon. We aimed at rearing the fish in either *r-* or *K*-selected environments, where the *r*-selected environment would be expected to be dominated by fast-growing opportunistic bacteria and thus represent more detrimental microbial environment than the *K*-selected water. Eggs from both wild and aquaculture strains of Atlantic salmon were hatched under germ-free conditions. One week after hatching, rearing flasks were inoculated with either *r-* or *K-*selected water communities. Three weeks after hatching, no effect of host strain on the gut microbiomes were observed. *r-*selection was found to take place in the rearing water of all flasks, including in the flasks added K-selected water. Still, the water microbiomes differed significantly between the flasks that had been added *r-* and *K-*selected water (Add-r and Add-K flasks, respectively). Lower alpha diversity and higher abundances of *Pseudomonas* were observed for the Add-K flasks, indicating a potential unfavorable microbial environment. Selection in the host structured the gut microbiomes, but an extensive interindividual variation was explained by stochastic processes in community assembly. The gut microbiomes also differed significantly between the Add-r and Add-K flasks. In Add-K flasks, they had higher similarities to the rearing water microbiomes, and the assembly of gut communities was less influenced by stochastic processes. The fish in Add-K flasks had lower growth rates than in Add-r flasks, probably as a result of negative host–microbe interactions. These findings highlight the importance of, but also the challenges related to, managing the microbial environment when cultivating fish.

## Teaser

The early gut microbiome of yolk-sac fry of Atlantic salmon is influenced by the microbial rearing environment, but not host strain.

## Introduction

Fish live in close interaction with environmental bacteria. Upon hatching, the exposed mucosal surfaces of the fish are rapidly colonized. Environmental bacteria are believed to be the most important source for the early fish microbiomes. The chorion microbiomes may also represent an important source of bacteria (Egerton et al. 2018), but their role in the initial colonization remains to be clarified. After mouth opening, the digestive tract is available for bacterial colonization. Building on Vellend’s ecological theory for community assembly (Vellend 2010, Nemergut et al. 2013) suggested that microbial community assembly can be explained by four fundamental ecological processes: diversification, dispersal, selection, and ecological drift (due to stochastic events). Of these, the three latter ones are most relevant for the development of the early fish gut microbiome (Burns et al. 2016, 2017, Vestrum et al. 2020).

The selection regime in the gut of a juvenile fish is influenced by a variety of factors, such as microbial interactions, host genetics, developmental stage (Bakke et al. 2015, Bledsoe et al. 2016, Yan et al. 2016, Zhang et al. 2018), and diet (Ringo et al. 2016, Michl et al. 2017). Although the microbiomes of fish larva appear to be highly distinct from those in the environments, there are indications that the composition of the water microbiomes affect the fish microbiomes (Bakke et al. 2015, Giatsis et al. 2015, Vestrum et al. 2020). The role of host genetics as a determinant for fish gut microbiomes is unclear. Li et al. (2018) found indications that host genetics does have an effect, while others found no or minor influence of host genetics on the gut microbiome of fish (Bledsoe et al. 2018, Dvergedal et al. 2020). Youngblut et al. (2019) examined the role of host evolutionary history on the diversity of gut microbiomes for phylogenetically divergent vertebrates and concluded that microbiomes of ray-finned fish generally seem to be little influenced by host phylogeny. Kim et al. (2021) found that host habitat was a more important determinant for the gut microbiome than host taxonomy and trophic level in a study that included 85 fish species.

Atlantic salmon is an anadromous species, spending its first years in rivers, before migrating to the sea. They return to their home river to spawn after a few years. After spawning and fertilization, which takes place from late autumn to mid-winter, the eggs are buried in coarse gravel, where they hatch during late spring (Saltveit and Brabrand 2013). In aquaculture hatcheries for Atlantic salmon, the eggs are incubated at high densities and usually disinfected prior to hatching. The hatching takes place at high egg densities, in systems that have higher organic loads and bacterial densities than under hatching in the natural river environments. The potential impact of these distinct microbial conditions on the early fish microbiomes and the fry health is not known. Moreover, the environments in the hatcheries are probably compatible with *r-*selection, and are expected to promote the growth of fast-growing, opportunistic bacteria, which may have detrimental effects on the newly hatched fish larvae (De Schryver and Vadstein 2014, Vadstein et al. 2018). According to ecological theory, *K-*strategists dominate in environments with low nutrient supply and bacterial densities close to the carrying capacity (Andrews and Harris 1986). Research suggests that aquaculture systems that are operated to maintain *K-*selected microbial environments counteract negative host–microbe interactions (Skjermo et al. 1997, Attramadal et al. 2014, Vadstein et al. 2018).

At hatching, Atlantic salmon possess a relatively large yolk. Koss and Bromage (1990) found that yolk sac fry reached their maximum wet weight around 435 degree-days posthatching, and the complete consumption of the yolk occurred some days after this. A study of the ontogeny of the digestive system of Atlantic salmon showed that at 7 days posthatching (dph), the mouth and anus was open, and the digestive tract was morphologically distinct with an early stomach, distinguishable proximal and distal regions, a rectum, as well as liver and pancreas (Sahlmann et al. 2015). The long yolk sac stage makes Atlantic salmon well-suited for studying the early community assembly of fish microbiomes and host–microbe interactions in the absence of external feeding (Canny et al. 2023).

In the present study, we characterized the early gut microbiome of yolk sac fry of a wild and an aquaculture strain of Atlantic salmon. We aimed to understand how the host strains, the microbial environment, and ecological processes affect the composition of the early gut microbiome. We used a two-factor factorial design experiment, with genotype (wild versus aquaculture salmon strains) and source water microbiomes (*r-* versus *K-*selected microbial communities) as the two factors.

## Methods

The fish experiment, which this study is based on was conducted as part of a master thesis, and the experiment and preliminary results are described in this thesis (Mathisen 2019).

### Fish rearing

Fertilized eggs from a wild strain of Atlantic salmon (the Rauma-1 strain) were delivered from Haukvik Genbank AS (Vinjeøra, Norway), and eggs from an aquaculture strain were delivered by AquaGen AS (Kyrksæterøra, Norway). The eggs from both strains had similar expected hatching days.

Germ-free Atlantic salmon fry were generated as described Canny et al. (2023). In brief, the eggs were incubated in an antibiotic cocktail containing ampicillin, amphotericin B, erythromycin, kanamycin, oxolinic acid, penicillin, and rifampicin [for details, see Canny et al. (2023)] for 24 h and then in a Buffodine™ (an iodine-based disinfectant; FishTech) solution (50 mg l^−1^) for 30 min. Thereafter, they were distributed into sterile tissue culture flasks (15–18 eggs per flask) using sterile technique. Eggs were reared in salmon gnotobiotic medium (SGM) in a volume of 100 ml. To maintain good water quality, 60% of the SGM was exchanged with either r- or K-selected water (water treatment described below) three times a week. After hatching, egg debris and dead eggs were carefully removed at water exchanges. The fish were reared in the dark at 7°C. The fish survival was registered every day of water exchange through the whole experiment. The body length of six fish randomly sampled form each from each of the 16 rearing flasks was measured at two time-points, 1 week after exposure to bacteria (15 dph) and at the end of the experiment (22 dph). This was done by euthanizing the fish in sterile ethyl 3-aminobenzoate methane sulfonate (5.2 g l^−1^; Sigma) before measuring the length from tip of snout to the end of notochord excluding caudal fin. Thus, at 15 dph, the number of individuals per flask was reduced by six.

### Experimental design

We used a two-factor factorial design experimental to investigate the effects of genetic strain of the salmon (a wild strain and an aquaculture strain) and microbial water quality (*r-* or *K-*selected water communities added to flasks) on the gut microbiomes of the salmon yolk sac fry. Until 7 dph, all flasks were reared under identical germ-free conditions with SGM as described by Gómez de la Torre Canny et al. (2023). The hatching day was defined as the day when at least 75% of the fish had hatched in all fish flasks. From seven dph, the fish were exposed to bacteria through addition of nonsterile freshwater. This water, added during the water exchanges three times per week, was treated to represent either *r-* or *K-*selected microbial communities (for details about the water treatment, see below). The design included four experimental groups: flasks with (1) wild salmon strain added *r-*selected water, (2) wild salmon strain added *K-*selected water, (3) aquaculture strain added *r-*selected water, and (4) aquaculture strain added *K-*selected water. For each experimental group, there were four replicate flasks. The experiment was maintained until 22 dph. We refer to flasks added *r-*selected water upon water exchanges as Add-r flasks, and flasks added *K-*selected water as Add-K flasks.

### Water treatment

The freshwater used in this experiment was supplied by Vikelvdalen drinking water treatment facility in Trondheim, Norway, and represented untreated water collected from the Lake Jonsvatnet (63.3655° N 10.5820° E) at a depth of 70 m. The water was prefiltered (1 µm) to remove larger particles and eukaryotic organisms and stored in 20 l containers at 7°C in the dark. Sterile freshwater was prepared by sterile filtering (0.2 µm) and autoclavation. This sterile water was used for rearing the germ-free fish (until 7 dph) and to dilute the *r-*selected water (explained below) from 7 dph. Prior to each water exchange, the collected lake water was used to produce two distinct microbial water qualities, representing *r-* and *K-*selected microbial communities. To produce water representing *r-*selected communities, 300 ml of lake water was given a nutrient pulse of 15 ml M65 nutrient solution (yeast extract, bacteriological peptone, and tryptone; 50 µg ml^−1^ of each) and incubated in darkness at room temperature for 24 h to allow for bacterial growth. For *K-*selected water communities, 300 ml of the untreated lake water was transferred to a flask and incubated at room temperature for 24 h. For each new round of water exchange, the bacterial density in the *r-* and *K-*selected water was assessed by flow cytometry (see below). To maintain approximately the same bacterial density in the *r-* and *K-*selected water to be added to the fish flasks, the *r-*selected water was diluted with sterile lake water to obtain a similar density as found for the *K-*selected water. The dilution factor varied between 20 and 50 among the days for water exchanges.

### Flow cytometry analysis

Flow cytometry was applied to determine the bacterial density in the water. Samples were collected upon water exchanges (three times a week). For the rearing water (RW) and for the added *r-* and *K-*selected water, around 40 ml were sampled in sterile 50 ml tubes and stored for maximum 6 h at 4°C until analysis. Prior to the analysis, samples were diluted in 0.1x TE-buffer to get a bacterial density below 1000 cells µl^−1^. A working solution of SYBR^®^ Green I nucleic acid gel stain (Life Technologies, Thermo Fisher Scientific Inc.) was added to 1 ml of the diluted samples to give a final concentration of 2x SYBR Green I and incubated in darkness at room temperature for 15 min. The samples were analyzed on a BD Accuri™ C6 Flow Cytometer (BD Bioscience, San Jose) with medium flow rate (34.5 µl min^−1^) for 2 min or until 10 000 events were collected. The results were processed using BD Accuri™ C6 Software. FL1-A (533 ± 15 nm) versus FSC-A (forward scatter, corresponding with the size of the cell) was plotted to filter out the noise and larger particles. The data were imported to Microsoft Excel for further analysis to estimate the bacterial density of the added water (AW) and RW. Flow cytometry data (.fcs files) are available at https://figshare.com/projects/Flow_Cytometry_Data/211171.

### Sampling of water and guts for microbial community analysis

Water samples for microbial community analysis were collected from each of the 16 fish flasks on 22 dph for the RW, and on 14, 16, 18, and 21 dph for the *r-* and *K-*selected water prior to addition to fish flasks. Water samples were filtered through a 0.2-µm syringe tip filter (DynaGard^®^) using a 20-ml syringe (around 10 ml or until the filter was clogged). The filters were stored at −20°C until further analysis. Gut samples for microbial community analysis were collected on 22 dph after euthanasia of four individuals per flasks (a total of 64 individual guts). The dissection was performed under a microscope under aseptic conditions. The yolk sac was removed, and the gut was gently pulled out and transferred into a cryotube. All equipment was cleaned with 70% ethanol between individuals. The samples were stored at −80°C until further analysis.

### DNA extraction, PCR, amplicon library preparations, and Illumina sequencing

The ZymoBIOMICS™ DNA Miniprep Kit (Zymo Research) was used to extract DNA from gut and water samples according to the manufacturer’s protocol with the following modifications: for the lysis step, shaking was performed using a vortex adapter for 2 ml tubes at maximum speed for 30 min. Elution volume for all extractions was 100 µl.

Amplicons of the variable regions 3 and 4 (V3 and V4) of the 16S rRNA gene were generated using the primer pair Ill338F (5′-TCGTCGGCAGCGTCAGATGTGTATAAGAGACAGNNNN**CCTACGGGWGGCAGCAG**-3′) and Ill805R (5′-GTCTCGTGGGCTCGGAGATGTGTATAAGAGACAGNNNN**GACTACNVGGGTATCTAAKCC**-3′), where target sequences are shown in bold. Polymerase chain reactions (PCRs) for the salmon gut samples were run for 38 cycles (98°C 15 s, 55°C 20 s, and 72°C 20 s) with reaction mixture with 1x Phusion buffer HF (Thermo Scientific), 0.3 µM of each primer (Sigma-Aldrich), 0.2 µM of each dNTP (Thermo Scientific), 0.025 U µl^−1^ Phusion Hot Start DNA Polymerase (Thermo Scientific), and 2 µl of a 1:10 dilution of the DNA extracts as template. The water samples were amplified using the same PCR conditions, but with 35 cycles, and 1 µl of undiluted DNA extracts as templates. The amplicons were normalized and purified using a Sequal Prep Normalization Plate Kit (Invitrogen) by following the manufacturers protocol. In a second PCR, each amplicon was indexed with a unique indexing primer pair by using the Nextera XT Index Kit Set D (Illumina, San Diego, CA). Indexing primers (2.5 µl each) were added to a PCR reaction mixture consisting of 1x Phusion buffer HF (Thermo Scientific), 0.25 mM dNTP (Thermo Scientific), and 0.015 U µl^−1^ Phusion Hot Start DNA polymerase (Thermo Scientific). PCRs were run for 10 cycles (98°C 15 s, 55°C 20 s, and 72°C 20 s). For the amplicons with low yield, a new indexing PCR with 12 cycles was run. For each of the indexed PCR products, 10 µl were normalized and purified using the Sequal Prep Normalization Plate Kit (Invitrogen) as described above. All indexed, normalized amplicons were then pooled together and concentrated by using Amicon Ultra 0.5 Centrifugal Filter units (Merck Millipore, Ireland). The concentration and purity of the concentrated sample were measured with NanoDrop™ One (Thermo Scientific). The amplicon library represented 64 individual gut and 16 RW samples from 22 dph, in addition to samples of the *r-* and *K-*selected lake water prior to addition to fish flasks (from 14, 16, 18, and 21 dph, a total of 8 samples). Negative controls for the DNA extraction and PCR were included for all PCR setups. The amplicon library was sent to the Norwegian Sequencing Centre for paired-end sequencing on one MiSeq run (Illumina) with V3 reagents (Illumina). The resulting amplicon sequencing data are deposited at the European Nucleotide Archive (accession numbers ERS17698177–ERS17698264).

### Processing of the illumina sequencing dataset and statistical analysis

The Illumina sequencing data were processed using the USEARCH pipeline (version 10; https://www.drive5.com/usearch/). The command Fastq_mergepairs was used for merging of paired reads, trimming off primer sequences, and filtering out reads shorter than 400 bp. The processing further included demultiplexing and quality trimming (the Fastq_filter command with an expected error threshold of 1). Sequences were further sorted by size and singleton reads were removed (fastx_uniques_sortbysize). A zero radius operational taxonomic unit [zOTU; referred to as amplicon sequencing variants (ASVs) in this manuscript] table was made by denoising using the Unoise3 command (Edgar 2016a). As recommended in the Usearch documentation, zOTUs with abundances lower than 8 reads in all samples were removed. Taxonomy assignment was performed applying the Sintax script (Edgar 2016b) with a confidence value threshold of 0.8 and the Ribosomal Database Projects reference data set v16 (Cole et al. 2014).

The resulting ASV table was manually inspected for further quality assessment, and a small number of nonabundant ASVs representing salmon genes were excluded from the table. Furthermore, all ASVs observed for the nontemplate controls and the negative controls for the DNA extraction protocol, were excluded. An exception was ASVs that were abundant in gut and water samples but had very low abundances in the negative controls (<4 reads in the negative controls). The resulting ASV table was normalized to 11 000 reads per sample (the lowest number of reads observed for any of the samples) by first determining the fraction of the ASVs for each sample, and then multiply with the relevant number of reads, and finally rounding off the read numbers to integers.

R (version 4.1.2) was used to analyze the amplicon sequencing data. All scripts are available at https://github.com/madeleine-gundersen/AtlanticSalmon_communityassembly. The alpha-diversity measurements observed ASV richness (S), Chao1, and Hill’s diversity index of order one (Hill 1973), corresponding to the exponential Shannon diversity (e^H^) were calculated using the functions reyni() and estimateR() from the vegan package (version 2.6–4). We calculated evenness as the exponential Shannon diversity divided by the ASV richness (e^H^/S). The Bray–Curtis similarity was used for comparison of community profiles within and between groups of samples (Bray and Curtis 1957). PCoA plots were generated using the function ordinate() and plot_ordination() from the R package phyloseq (version 1.41.1). All plots were generated using functions from ggplot2 (version 3.4.4). If not otherwise is stated, all PCoA and PERMANOVA tests were based on Bray–Curtis similarities. SIMPER (Similarity Percentage) analysis based on Bray–Curtis similarities were conducted to identify the ASVs contributing to differences in the microbial community composition between sample groups (Clarke 1993) by using PAST (Hammer et al. 2001).

We applied two-way ANOVA in the program package PAST v. 4.17 (Hammer et al. 2001) to test whether body lengths were impacted by salmon strain or water treatment. Normality of the residuals was confirmed with the Shapiro–Wilk test using the stats package in R v4.2.3. A two-sample *t*-test was used to test whether alpha diversity indices and Bray–Curtis similarities differed significantly between two sample groups. We tested that data were normal distributed and had equal variance. If the variance between samples was significantly different (F-test, *P* < .05), an unequal variance *t*-test (Welch *t*-test) was used. If the data were not normally distributed, we used a Wilcoxon test.

To infer the ecological processes contributing to the community assembly in the fish and its RW we used two null-model approaches, the nearest taxon index (NTI) and beta nearest taxon index (βNTI). Both of these null models use a phylogenetic tree. Thus, we first generated a phylogenetic tree. The ASV sequences were aligned using the function AlignSeqs() from the DECIPHER package (version 2.22.0). The Phangorn package (version 2.8.1) was then used to construct a phylogenetic tree. We constructed a neighbor*-*joining tree and then optimized the tree model parameters to a generalized time-reversible model with the gamma rate variation to produce a maximum likelihood tree. The phylogenetic tree was rooted at the branch with the longest length using the package Ape (version 5.6–2). Next, a phylogenetic distance matrix was calculated using the function cophenetic() from stats (version 4.1.2). The underlying assumption for the null models is that there is a phylogenetic signal in the community, and we quantified this signal using the function mantel.correlog() from vegan.

NTI assesses assembly at the sample level (Webb et al. 2002) and quantifies if members of the community are more or less phylogenetically clustered than expected by chance. To assess this, a randomly generated null-community was generated from the metacommunity by randomly shuffling the taxa on the phylogenetic tree tips. An NTI > 2 indicates that the community is more clustered than expected by chance, i.e. that selective processes such as environmental filtering has structured the community. An NTI←2 indicates phylogenetic overdispersion, i.e. that communities are under different selective pressures. An NTI that is not significantly different from the null model (i.e. |NTI| < 2) indicate that stochastic processes are structuring the community. The NTI was estimated for all gut and RW samples at 22 dph. Since all ASVs originated from the same lake water, we regarded the whole dataset as one metacommunity. NTI was estimated using the function ses.mntd(null.model=“taxa.labels”, runs = 999) from the package Picante (version 1.8.2).

Next, the βNTI index was estimated to evaluate the community assembly processes structuring between-sample differences. βNTI reflects the phylogenetic dissimilarity between samples. Following Stegen et al. (2015), for a single between-sample comparison, a βNTI > 2 indicates that the phylogenetic turnover is greater than expected by chance (i.e. compared to a null model) and that heterogeneous selection is structuring the community assembly. In contrast, for βNTI < −2, homogeneous selection is assumed to dominate community assembly. For comparisons not significantly different from the null model, the community assembly is driven by stochastic processes, such as drift. βNTI was estimated separately for four sample groups using the function qpen() from the package iCAMP (version 1.5.4): RW samples from Add-K flasks, RW samples from Add-r flasks, gut samples from Add-K flasks, and gut samples from Add-r flasks. NTI and βNTI estimates were plotted using the package ggplot2 (version 3.3.5).

## Results

### Alpha diversity of gut and water microbiomes

**Figure 1.**
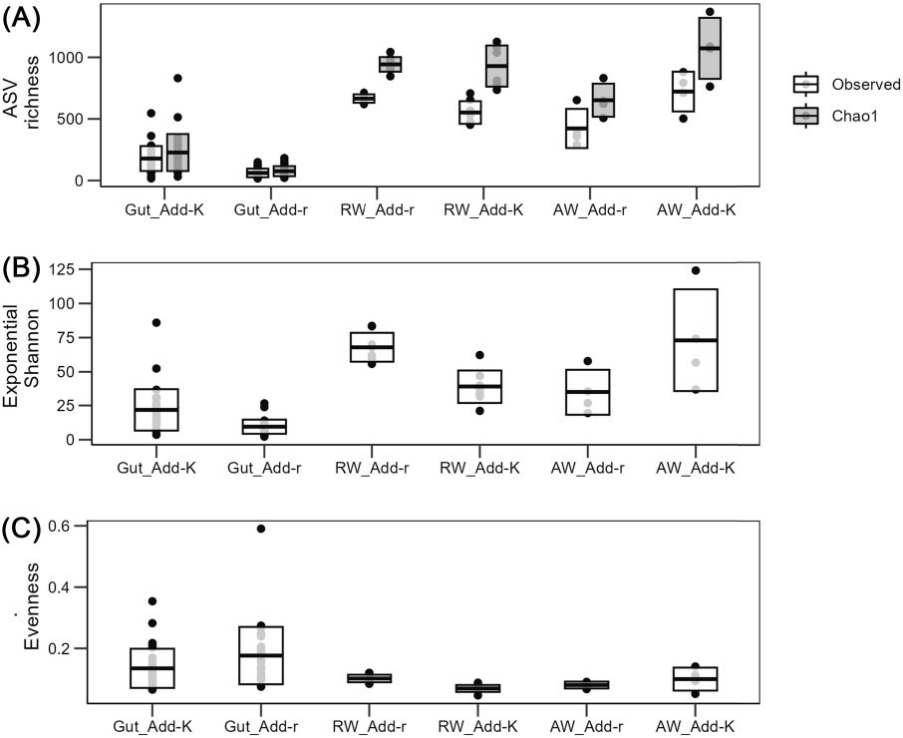
Alpha diversity indices for gut samples, RW samples, and samples of the water added to fish flasks at water exchanges. (A) Observed and Chao1 ASV richness, (B) exponential Shannon diversity, and C) evenness. Each dot represents one sample. Add-K and Add-r indicates flask added *K-* and *r-*selected water upon water exchanges, respectively, and includes both flasks with the aquaculture and the wild salmon strain. There were eight rearing flasks per group (four for each of the wild and aquaculture strain of salmon), and individual guts were sampled for four individuals per flasks. RW samples (one per replicate flask; in total 16 flasks) and gut samples were collected on 22 dph, and samples of the *r-* and *K-*selected water prior to addition to the rearing flasks were collected 14, 16, 18, and 21 dph. RW: rearing water; AW: added water.

In total, 3149 ASVs were observed in all samples after a strict quality filtering of the ASV table (for details, see the section “Methods”). The ratio between the observed ASV richness and the Chao1 richness indicate that on average around 80% and 65% of the theoretical richness was covered by the sequencing effort for gut and water samples, respectively. For microbiomes associated with the water that was added to the rearing flasks at the water exchanges (i.e. *prior* to the water exchange), the ASV richness, the exponential Shannon’s diversity, and the evenness were higher for the *K-*selected water compared to the *r-*selected water (Fig. 1), although the difference was significant only for the ASV richness (*t*-test; *P* = .04). Surprisingly, an opposite trend was observed for the RW samples: the alpha-diversity (ASV richness, the exponential Shannon’s diversity index, and the evenness) was significantly higher for samples taken from the Add-r flasks (Fig. 1; Welch *t*-test, *P* < .005). For the gut microbiomes, the ASV richness and exponential Shannon diversity was higher for samples from the Add-K flasks compared to Add-r flasks (Fig. 1; Wilcoxon *P* < .001), but the evenness was significantly higher for gut samples from the Add-r flasks (Fig. 1; Wilcoxon, *P* = .04).

### Effect of water treatment on the water microbiomes prior to addition to rearing flasks

**Figure 2.**
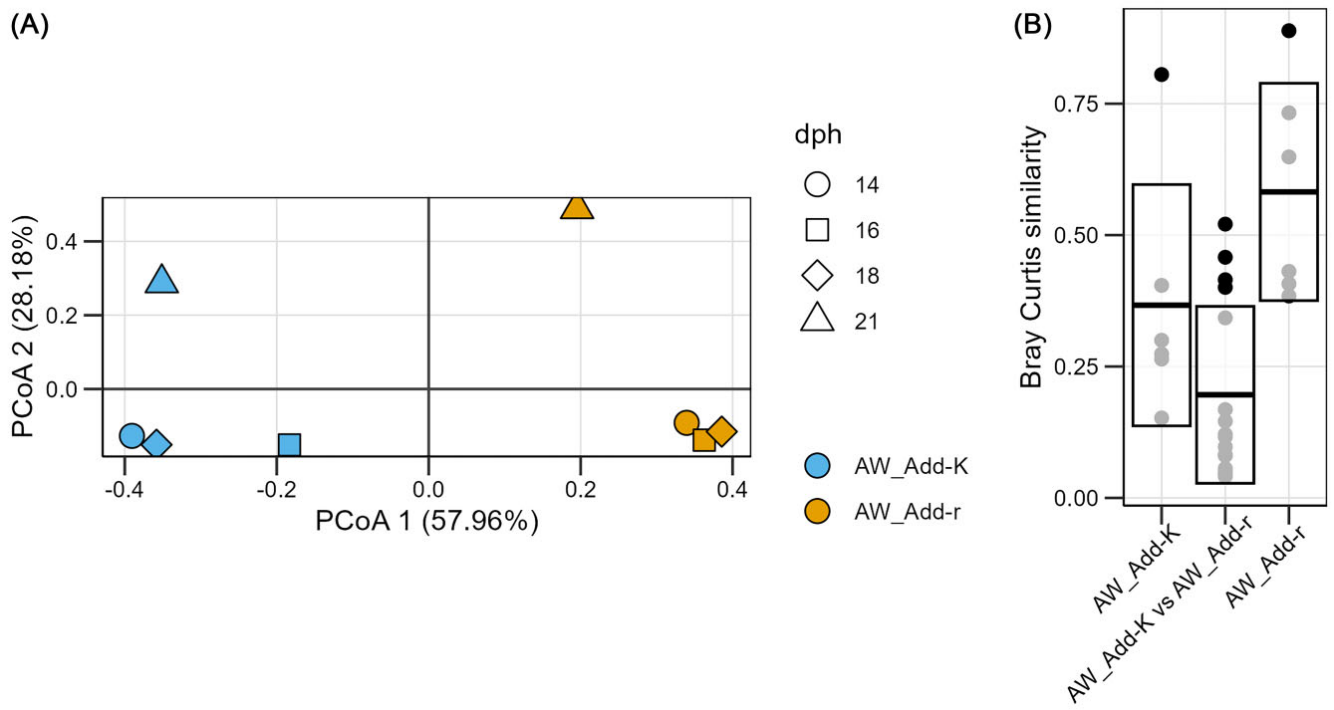
Comparison of the microbiomes associated with the *r-* and *K-*selected water that was added to fish flasks. Samples were collected on 14, 16, 18, and 21 dph. (A) A PCoA ordination based on Bray–Curtis similarities. For each treatment (i.e. addition of either *r-* or *K-*selected to flasks) one sample was collected on each sampling time. (B) Bray–Curtis similarities for comparisons of the composition of microbiomes within and between treatments. Each point represents a comparison between two samples. Add-r and Add-K indicate water treatment, i.e. addition of either *r-* or *K-*selected water, respectively.

We characterized the microbiomes associated with the *r-* and *K-*selected water qualities that were added to the rearing flasks upon water exchanges on 14, 16, 18, and 21 dph. A PCoA ordination based on Bray–Curtis similarities (Fig. 2A) and Bray–Curtis similarities for comparison of community composition between the treatment groups (Fig. 2B) indicated that the community composition differed between the *r-* and *K-*selected water prior to the addition to the rearing flasks. A one-way PERMANOVA showed that the difference was significant (*P* = .03).

**Figure 3.**
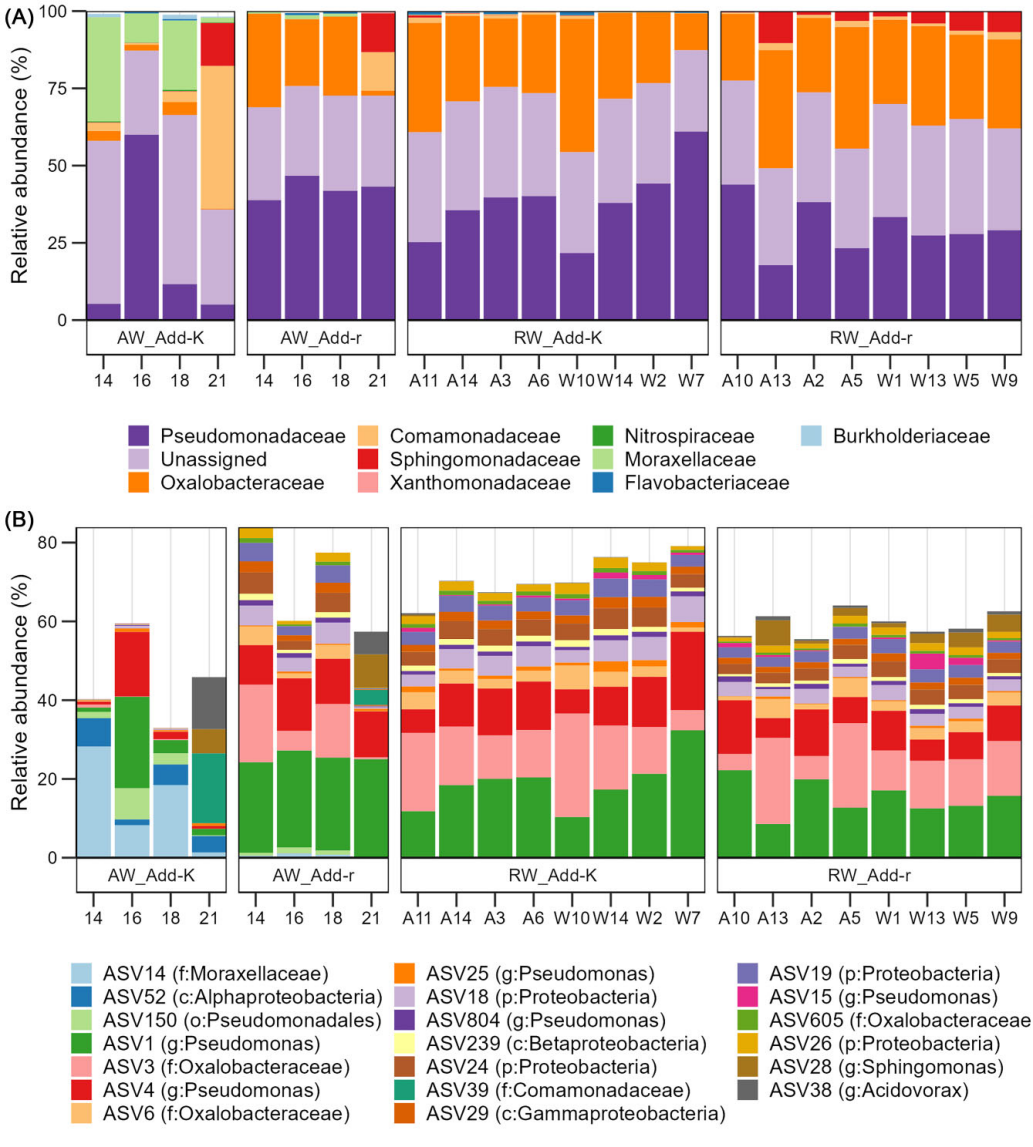
Community composition for samples of the water added to fish flasks and the RW. (A) Community composition at the family level. Only families with relative abundances higher than 1% in at least one sample were included. (B) Relative abundance of the 20 most abundant ASVs. For the samples of the AW (i.e. the water added to the rearing flasks at water exchanges), the number given under the bars for each sample indicates the sampling day (14, 16, 18, and 21 dph). For the RW, one sample per rearing flask was collected on 22 dph. For the RW samples, W designate samples from flasks with fry of the wild salmon strain, while A designate samples from flasks with the aquaculture salmon strain, and numbers indicate the rearing flask.

The community composition clearly differed between added the *r-* and *K-*selected water, both at the family level and the ASV level (Fig. 3A and B, respectively). For the *r-*selected water, *Pseudomonadeceae* and *Oxalobacteraceae*, represented by a few ASVs, dominated, and accounted for around 60% of all reads at most sampling times (Fig. 3A). For the *K-*selected water, the community composition varied more between sampling days. *Pseudomonadeceae* and *Oxalobactereae* were less dominating, and *Moraxellaceae* was more abundant (Fig. 3A). On 21 dph, three ASVs representing *Comamonadaceae, Sphingomonas*, and *Acidovorax* had high relative abundances in the *K*-selected water, but also in the *r*-selected water, although lower (Fig. 3B). This variation was also reflected in Bray–Curtis similarities, which indicated that the microbiomes of the *K-*selected water changed more over time than the *r-*selected water (Fig. 2B). Together with the alpha-diversity analysis (Fig. 1), these results suggest that the *r-* and *K-*selection regimes created distinct bacterial communities, and that the water microbiomes added to the fish flasks during water exchanges were in fact *r-* and *K-*selected.

### The microbiomes of the RW

**Figure 4.**
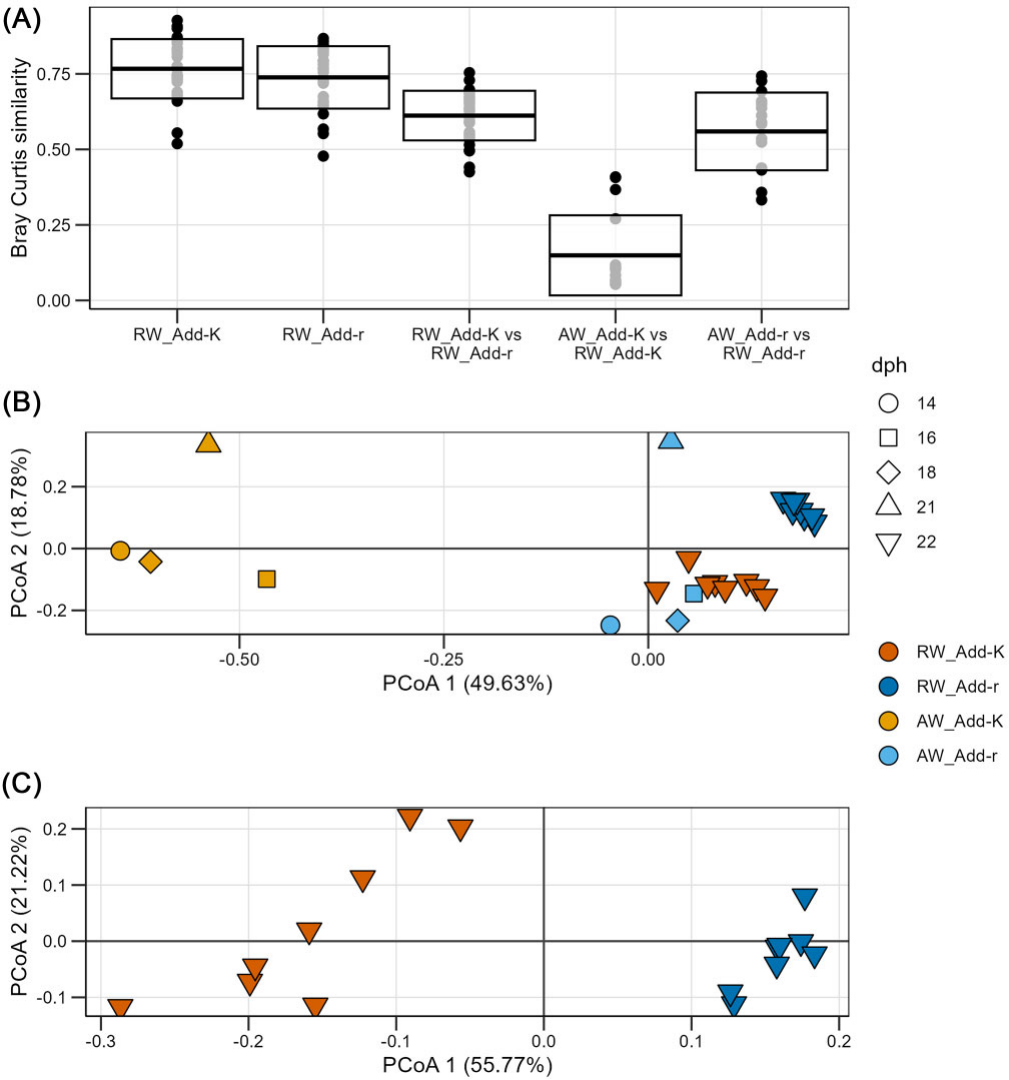
Comparison of the microbiomes associated with the RW and the AW. (A) Bray–Curtis similarities for comparison of community composition of *K-* and *r-*selected AW (i.e. the water added to the rearing flasks at water exchanges) and the RW. (B) A PCoA plot based on Bray–Curtis similarities for comparison of community composition between samples of the AW and the RW. (C) A PCoA plot based on Bray–Curtis similarity for comparison of the composition of RW microbiomes between Add-r and Add-K flasks. Each point represents one sample. For each rearing condition (Add-K and Add-r), there were eight replicate rearing flasks, including flasks both the wild and aquaculture strain of salmon. RW samples (one per flask; in total 16 flasks) were collected 22 dph, and samples of the water added to the rearing flasks were collected 14, 16, 18, and 21 dph.

Even though the microbiomes of the water added to the Add-r and Add-K flasks differed, the community composition at the family level of the RW microbiomes were relatively similar on 22 dph (Fig. 3A). *Pseudomonadeceae* and *Oxalobactereae* were highly abundant in all RW samples, as in the *r-*selected water prior to addition to the fish flasks. Next, we compared the composition of the microbiomes of the AW to those of the RW. Interestingly, for the Add-r flasks, the similarity was high (average Bray–Curtis similarity of 0.55), whereas for Add-K flasks it was low, with an average Bray–Curtis similarity of 0.15 (Fig. 4A). This was also indicated by a PCoA ordination that showed that RW samples from both Add-r and Add-K flasks clustered together with samples of the *r-*selected water added to the Add-r flasks, but not with those of the *K-*selected AW (Fig. 4B). These observations indicate that the microbiomes in the RW were subjected to *r-*selection in the fish flasks, regardless of the community composition of the water added to the flasks.

At each water exchange, we examined the density of bacterial cells by flow cytometry both in the *r*- and *K*-selected water, prior to addition to rearing flasks, and in the RW exchanged from the rearing flasks. The cell densities varied between days and types of water samples but were always higher in the RW compared to the *r*- and *K-*selected (between 5 and 20 times from 7 dph; Fig. S1). This verifies that there was a substantial growth of bacteria in the RW.

**Figure 5.**
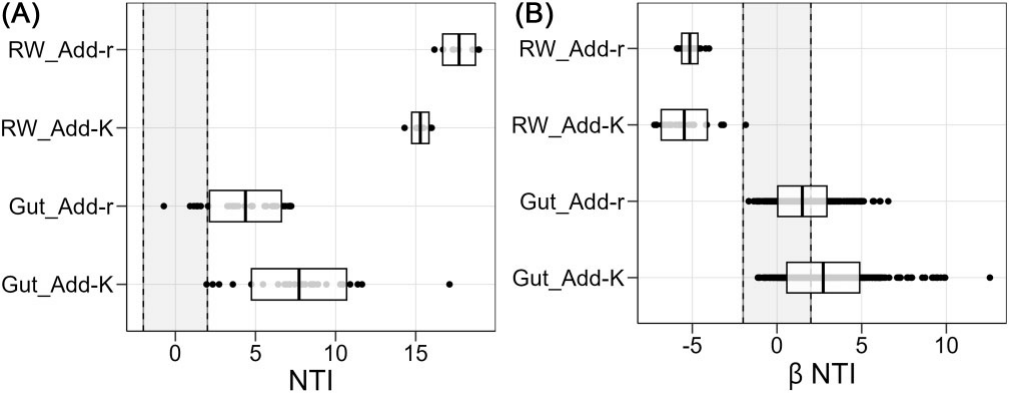
Community assembly processes in the gut and RW samples at 22 dph. (A) The NTI for each individual community in gut and RW samples. An NTI value larger than 2 indicates that the community is phylogenetically clustered, whereas values ←2 indicate phylogenetic overdispersion. Values between −2 and 2 reflect that communities are not significantly different from the null model. For the gut samples there were 32 samples per group (Add-r and Add-K), and for RW samples there were eight samples per group. (B) The βNTI for between sample comparisons within gut and RW samples for each rearing regime. βNTI > 2 indicates heterogeneous selection and βNTI < −2 indicates homogeneous selection. βNTI between −2 and 2 indicates that the community comparison is not significantly different from the null model. The solid black lines represent the mean and the boxes the standard deviation. Add-K: samples taken from flasks added *K-*selected water; Add-r: samples taken from flasks added *r-*selected water. For gut samples, *n* = 496 comparisons for each group, for RW samples, *n* = 28 comparisons for each group.

To infer the ecological processes contributing to the community assembly in the RW we performed NTI analysis. The microbiomes of the RW in both the Add-K and Add-r flasks were strongly phylogenetically clustered, with an average NTI of 15.3 for the Add-K samples and 17.7 for the Add-r samples (Fig. 5A). This indicates that selective processes structured the microbiomes of the RW, and that specific populations from the metacommunity were selected for in the RW. Furthermore, the community assembly in the RW was strongly dominated by homogeneous selection (i.e. βNTI < −2). As much as 96.4% and 100% of the comparisons among RW samples within the Add-K and Add-r sample groups, respectively, had a clearly more similar phylogenetically clustered patterned than expected by chance (Fig. 5B). These observations indicate that selection acted on the water microbiomes in all flasks.

To compare the composition of the RW microbiomes between the Add-r and Add-K flasks, we performed a PCoA. The resulting plot indicated that the samples clustered according to the treatment of the AW (Fig. 4C), and a PERMANOVA showed that the difference was significant (*P* = .0003). Despite this, three ASVs dominated the RW microbiomes in all flasks: ASV1 (*Pseudomonas*), ASV3 (*Oxalobacteraceae*), and ASV4 (*Pseudomonas*) (Fig. 3B). The same ASVs also dominated the microbiomes of the AW. They generally constituted as much as 40%–50% of the total reads for the RW samples (Fig. 3B). However, an ASV representing *Sphingomonas* (ASV28) was more abundant in the RW samples of the Add-r flasks, whereas ASV1 and ASV4, both representing *Pseudomonas*, were more abundant in the RW samples from the Add-K flasks (Fig. 3B).

### Gut microbiomes

**Figure 6.**
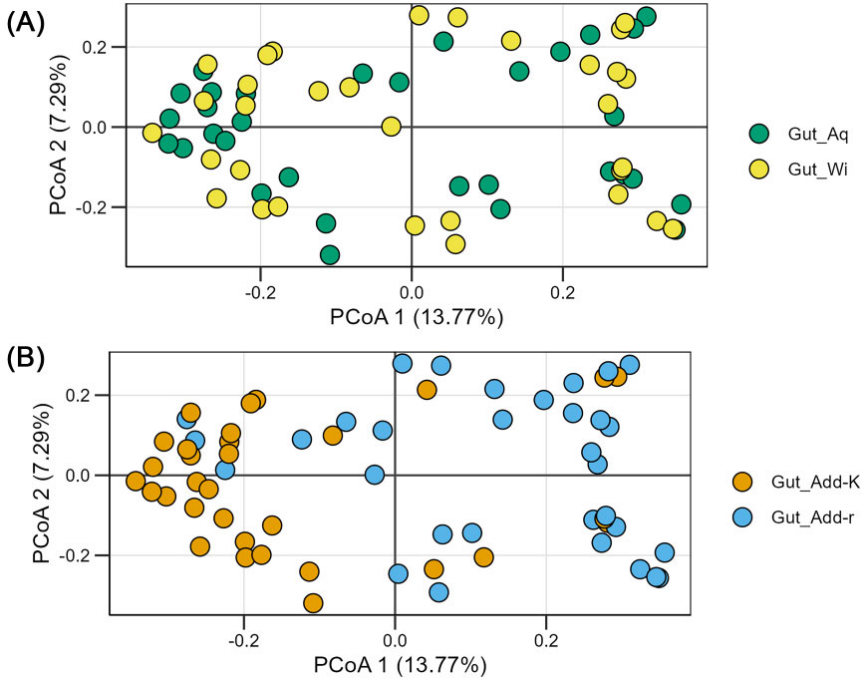
PCoA based on Bray–Curtis similarities for comparison of community composition among gut samples. (A) Samples labeled according to host strain; the wild strain (Wi) and aquaculture strain (Aq). (B) The same PCoA, but with samples labeled according to the type of water added to the flasks (Add-r and Add-K flasks). Samples represent single guts dissected from each of four individuals in each of four replicate flasks per condition at 22 dph.

To examine the influence of host strain and the treatment of the AW on the gut microbiomes, we performed a PCoA. No apparent clustering of gut samples according to salmon strain was observed (Fig. 6A), and a one-way PERMANOVA test did not show any significant difference in the composition of the gut microbiomes between the salmon strains. However, the same PCoA ordination indicated that the composition of the gut microbiomes differed between Add-K and Add-r flasks (Fig. 6B), and the differences were found to be significant (one-way PERMANOVA, *P* = .0001). Thus, the water microbiomes significantly affected the early colonization of the gut. In contrast, the host strain appeared to have little effect. In the subsequent analysis we therefore focused on the effect of the water communities on the gut microbiomes and treated all fish flasks that had received the same type of water as one experimental group, regardless of salmon strain.

**Figure 7.**
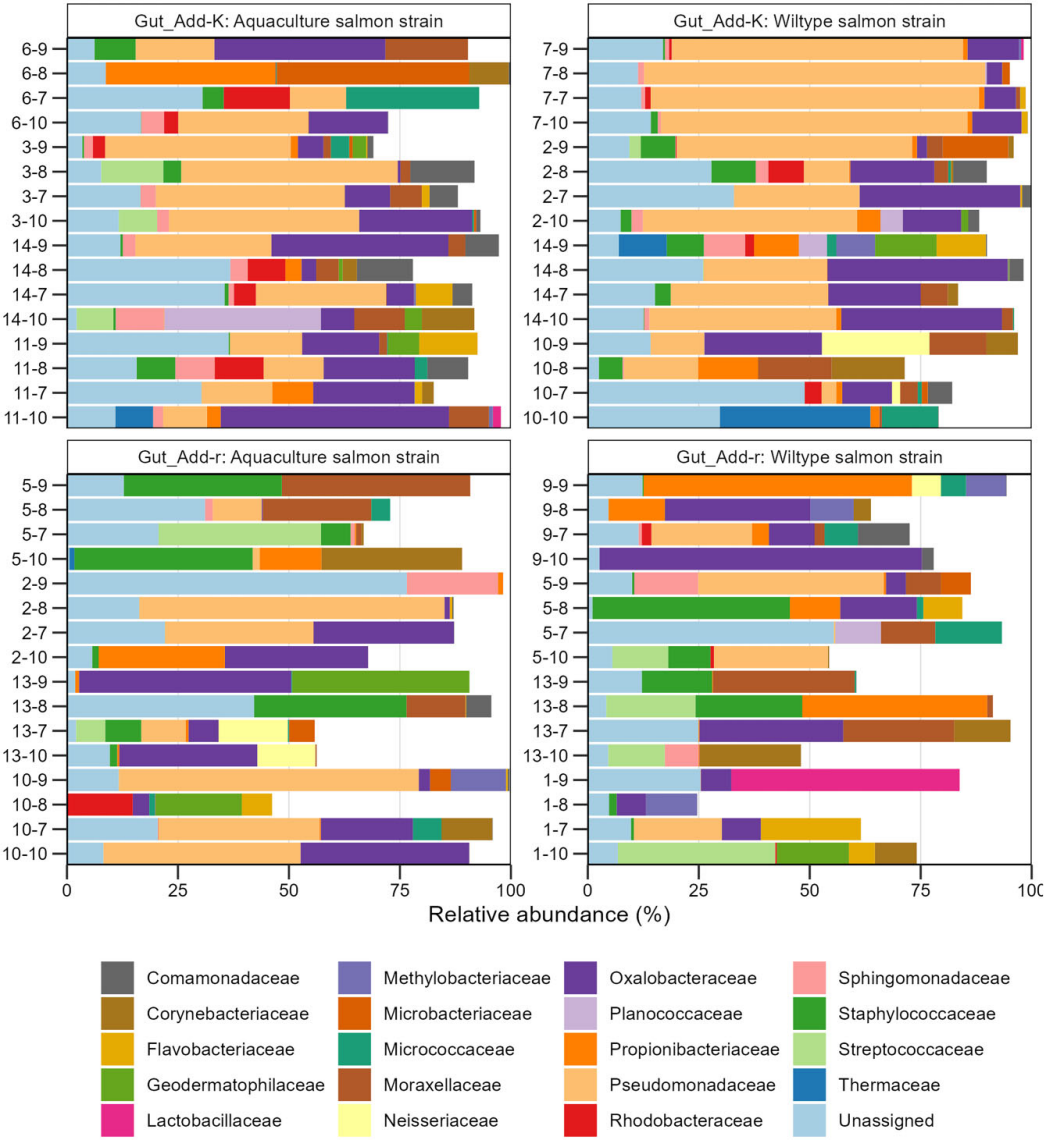
Community composition at the family level for gut samples. Four individual gut samples were collected from each rearing flask at 22 dph, and there were four replicate flasks for each rearing condition, i.e. Add-r and Add-K flasks with fry of either the wild or aquaculture salmon strain. Families with relative abundance below 10% in all samples a were not included. Each bar represents one sample. The numbers specified for each bar indicate the ID of the relevant rearing flask (first number) and the ID of the fish (second number).

The composition of the salmon gut microbiomes was highly variable between individuals at both the family and the ASV level, even for samples taken from the same rearing flask (Fig. 7, Fig. S3A). This was particularly profound for the gut samples from the Add-r flasks. Bray–Curtis similarities confirmed that the community composition varied extensively among individuals (Fig. S2), and significantly more for the gut microbiomes from the Add-r flasks (Welch *t*-test, *P* < .001; average Bray–Curtis similarity 0.025 compared to 0.090 the Add-K flasks).

Next, we performed NTI analysis to examine which ecological processes contributed to the community assembly of the gut microbiomes. This indicated that the gut microbiomes were more phylogenetically clustered than expected by chance, suggesting that populations from the metacommunity were selected for in the guts. However, the NTI values were lower than for the RW microbiomes; around 4 and 2 times for the Add-r and Add-K flasks, respectively (Fig. 5A). Moreover, gut microbiomes from Add-r flasks had lower NTI values than those from Add-K flasks (average NTI around 5 and 8, respectively), indicating a stronger impact of stochastic processes (Fig. 5A). We further applied the βNTI index to investigate which ecological processes were responsible for the high variation in community composition among gut samples (Fig. 5B). The results indicated that the phylogenetic turnover was greater than expected by chance, and that community assembly was mainly driven by stochasticity (41% and 68% for samples from Add-K and Add-r flasks, respectively) and heterogeneous selection (59% and 32% for samples from Add-K and Add-r flasks, respectively).

**Figure 8.**
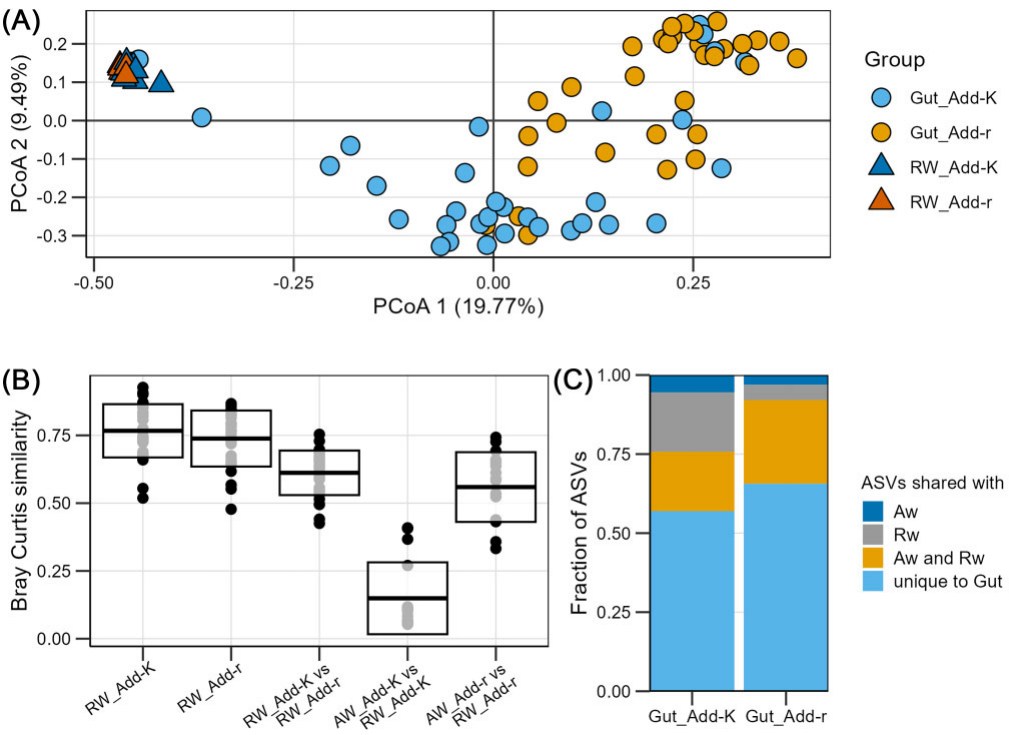
Comparison of microbiomes associated with gut samples, RW samples, and samples of water added to rearing flasks. RW samples (one per replicate flask; in total 16 flasks) and gut samples (four per rearing flask) were collected 22 dph, and samples of the water added to the rearing flasks were collected 14, 16, 18, and 21 dph. (A) A PCoA ordination based on Bray–Curtis similarities. Each point represents a sample. (B) Bray–Curtis similarities for comparisons of microbiomes of gut, RW, and AW samples. (C) Bar graph indicating the fraction of ASVs of the gut samples that were shared with the samples of the RW and the AW.

Finally, we compared the RW and gut microbiomes. A PERMANOVA test showed that the composition of the gut and RW microbiomes differed significantly both in the Add-K and Add-r flasks (*P* = .0002 and *P* = .0001, respectively). However, a PCoA ordination indicated that the gut and RW microbiomes were more similar to each other in the Add-K flasks compared to the Add-r flasks (Fig. 8A). This was confirmed by average Bray–Curtis similarities, which demonstrated that RW and gut microbiomes were almost four times more similar in the Add-K flasks than in the Add-r flasks (Fig. 8B). Many of the gut microbiomes from Add-K flasks had strikingly high abundances of two ASVs classified as *Pseudomonas* (ASV1 and ASV4; Fig. S3). Although their relative abundances varied extensively among individuals, they were on average 2.6 times more abundant in gut samples from Add-K flasks compared to Add-r flasks, with average relative abundances of 21.4% and 8.1% in Add-K and Add-r flasks, respectively (Fig. S3). As described above, these ASVs were also more abundant in the RW of the Add-K flasks, but only around 1.3 times higher than in the Add-r flasks (on average around 30% and 24% in Add-K and Add-r flasks, respectively). Moreover, the number of ASVs that were unique to the gut microbiomes was on average around 80% for samples from the Add-r flasks, but only 40% in the Add-K flasks (Fig. 8C). Taken together, these results suggest that the RW had a stronger influence on the gut microbiomes in the Add-K flasks.

### Fish survival and growth

**Figure 9.**
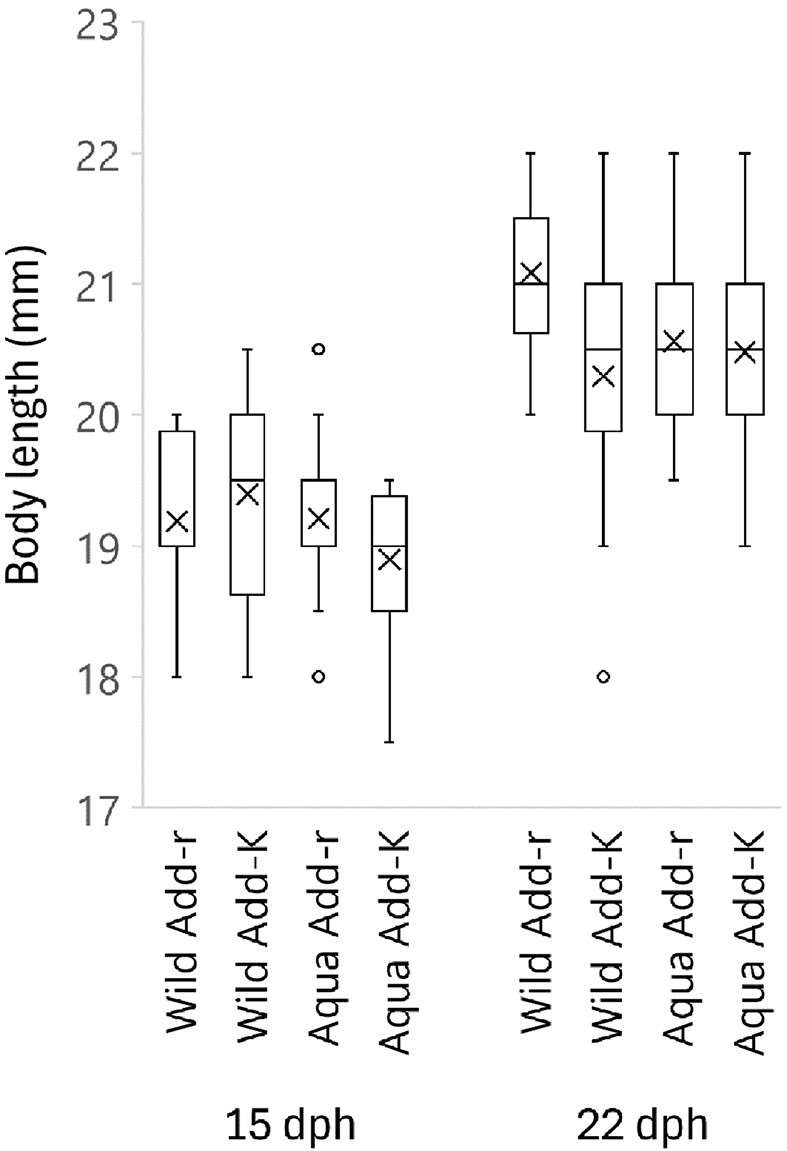
Body length of the yolk sac fry on 15 and 22 dph. On 15 and 22 dph, six individuals were collected from each of four replicate rearing flasks for each sample group. Thus, each bar represents 24 individuals. An exception was one of the Add-K Flasks rearing flask with only four individuals left on 22 dph (due to previous sampling and a few unhatched and dead eggs). Means are indicated by crosses. Add-K and Add-r indicates samples from flasks added *K-*selected water or *r-*selected water upon water exchanges, respectively. Wild and Aqua indicates samples from flasks with fish from the wild and aquaculture salmon strains, respectively.

The performance of the fish was assessed by registering mortality after the onset of exposure to bacteria on 7 dph and by measuring the body length as an indication of growth at 15 and 22 dph (Fig. 9). No fish mortality was registered for any of the rearing flasks. To investigate the potential impact of salmon strain or the microbial rearing environment (i.e. being reared in either Add-r or Add-K flasks) on fish growth, we performed a two-way ANOVA test of body lengths. On day 15, neither salmon strain nor the microbial rearing environment (Add-r or Add-K flasks) significantly affected the body length of the fish. For the body lengths measured on 22 dph, the test showed no significant difference between the wild and the aquaculture strains (*P* = .23). However, the fish reared in the Add-r flasks were significantly longer than of those reared in the Add-K flasks (*P* = .0057). Furthermore, an interaction effect was found (*P* = .025): The body lengths for the wild salmon strain appeared to be stronger influenced by the microbial rearing environment than the aquaculture strain (Fig. 9, Fig. S4).

## Discussion

In this study, we examined the gut microbiomes of yolk sac fry of Atlantic salmon only 3 weeks after hatching, i.e. only around 2 weeks after the opening of the digestive tract (Sahlmann et al. 2015). We aimed at clarifying the importance of host genetics (i.e. salmon strain) and the microbial environments for the composition of the early gut microbiome. In aquaculture, fish are typically exposed to microbial environments characterized by high and variable loads of organic matter which are expected to select for growth of opportunistic bacteria with high growth rates (De Schryver and Vadstein 2014). To which extent such microbial environments influences the microbial colonization of a newly hatched fish larvae and fish health and development, is poorly understood. We have previously advocated the importance of managing the microbial water quality in fish rearing to promote positive fish–microbe interactions and argued that this can be obtained through *K-*selection of the microbial communities suspended in the water (Skjermo et al. 1997, Attramadal et al. 2014, Vadstein et al. 2018). Here, we aimed to expose the fish to two distinct microbial environments: either *K-*selected or *r-*selected water microbiomes. We succeeded in generating *r-* and *K-*selected water communities that were added to fish flasks at water exchanges. Compared to the untreated lake water, the communities of the water that had been treated with a nutrient pulse showed typical indications of *r-*selection: low alpha diversity and high abundances of a few ASVs classified as *Pseudomonas* and *Oxalobacteraceae*. The genus *Pseudomonas* includes many opportunistic and specific pathogens (Yi and Dalpke 2022).

For the RW, however, we found that the microbiomes were subjected *r-*selection in all fish flasks. This was supported by the high bacterial densities in the RW compared to the lake water added on water exchanges, which indicate a potential for microbial growth in the flasks. Even though no fish feed was added to the flasks, the carrying capacity was obviously considerably higher than that of the added lake water. Egg debris and excretions from the fish are the most obvious sources of substrates for bacterial growth. Furthermore, the βNTI revealed a strong influence of homogenous selection for the water communities in all rearing flasks. This finding is compatible with *r-*selection acting on the water communities, although we cannot exclude the influence of other factors, such as for example microbial interactions. Still, the composition of the RW microbiomes differed significantly between the Add-r and Add-K flasks. In the Add-K flasks, the alpha diversity was lower, and the abundance of *Pseudomonas* was higher. Thus, the RW microbiomes of the Add-K flasks showed characteristics of *r-*selection to an even greater extent than those in the Add-r flasks. A potential explanation is that for the Add-r flasks, the *r-*selected AW already contained several bacterial populations representing *r-*strategic, rapid-growing bacteria, fit for the *r-*selected environment in the flasks. In the Add-K flasks, there was less competition from rapid-growing bacterial populations present in the water that was added to the flasks. These results demonstrate the challenge of creating a stable and *K-*selected microbial environment in fish rearing systems, unless the carrying capacity is similar for the input water and the RW.

Characterization of the gut microbiomes revealed several interesting findings. First, we did not observe effects of host strain on the early gut microbiomes in salmon. Youngblut et al. (2019) concluded that the gut microbiomes in ray-finned fish generally were little influenced by host phylogeny and argued that this can be explained by a close relationship to environmental, transiently present microbes. Still, other studies have found effects of host genetics on fish microbiomes (Li et al. 2018). In this experiment, we only investigated the very early gut microbiome. Potential effects of host genetics might be more profound at later developmental stages with exogenous feeding and when the intestine and the immune system are more developed. The host strains in this study might also be too little divergent to expect an observable effect on the gut microbiomes. However, the domestication and selective breeding of Atlantic salmon started in the early 1970s and the genetic divergence between wild and farmed Atlantic salmon strains is well documented (Karlsson et al. 2011).

Second, the large variation between gut communities of individuals was striking, even within rearing flasks, particularly in the Add-r flasks. Such high variation may be explained by stochastic processes in the community assembly (Zhou and Ning 2017), as suggested by the βNTI analysis. As all individuals within a treatment group were subjected to identical rearing and we expected similar selection regimes in the individual guts, stochastic processes were apparently the primary driver for the interindividual variation in gut microbiomes. The NTI analysis, on the other hand, indicated that selection in the gut structured the gut microbiomes. The fact that the NTI analysis indicated that selection in the gut structured microbiomes, whereas the βNTI suggested that stochastic processes were responsible for the high interindividual variation, may be explained as follows: during the bacterial colonization of the gut, only a subset of bacterial populations in the environment are able to colonize the fish gut, but which of these populations that are successful, is a result of stochastic processes. We only examined the early gut microbiome at one time point. Developmental changes of the intestine and the immune system may lead to an increased role of deterministic processes as the host matures. Burns et al. (2016) found that this was the case for developing zebrafish. Other studies, however, examining cod larval microbiomes (Vestrum et al. 2020), the freshwater species *Siniperca chuatsi*, and *Schizothorax meridionalis* (Yan et al. 2016), and zebrafish (Yan et al. 2012), indicated that the contribution from stochasticity on assembly of fish microbiomes increased with age. Studies with higher temporal resolution throughout the development are needed to clarify the role of stochastic processes in community assembly of gut microbiomes in fish.

Third, the gut microbiomes appeared to be more influenced by the RW microbiomes in the Add-K flasks than in the Add-r flasks. As explained above, the water microbiomes in the Add-K flasks showed indications of strong r-selection, even to a greater extent than the Add-r flasks. Compared to the Add-r flasks, gut microbiomes sampled from Add-K flasks were more similar to the RW microbiomes, and they had around 2.6 times higher relative abundances of two ASVs classified as *Pseudomonas* (ASV_1 and ASV_4). These ASVs were around 1.3 times higher in the RW of the Add-K flasks. Furthermore, the evenness was lower for gut microbiomes sampled from the Add-K flasks. High evenness has been found to be important for the multifunctionality of microbial ecosystems (Hillebrand et al. 2008, Zhang et al. 2021). Although the correlation between evenness and the health status of the human gut microbiome is not fully understood (Hagerty et al. 2020), reduced evenness has been observed during bacterial infections (Antharam et al. 2013), and for oysters, reduced evenness has been associated with increased susceptibility to Pacific Oyster Mortality Syndrome (Clerissi et al. 2020). The NTI analysis suggested that the gut microbiomes in the Add-K flasks were more influenced by deterministic processes. This determinism may be explained by a higher relative abundance in the RW of *Pseudomonas* populations that had better fitness in the fish gut, and therefore a competitive advantage compared to other bacterial populations during the early colonization of the fish guts.

Finally, the microbial rearing environment, but not the salmon strain, significantly influenced the body length of the fish only 2 weeks after the first exposure to bacteria. We previously demonstrated that cod larvae exposed to opportunistic bacteria in the RW showed upregulation of gene transcripts that were associated with pathogen recognition, infection, and immunity responses (Vestrum et al. 2018). A likely scenario is that stress imposed by negative fish–microbe interactions in the Add-K flasks reduced the growth of the fry. Interestingly, the growth of wild salmon strain appeared to be stronger influenced by the microbial rearing environment than the aquaculture strain. A general aim of breeding programs for aquaculture species is to improve robustness to infections (Johnston et al. 2024), and a possible explanation could be that the selective breeding of the aquaculture strain resulted in higher robustness to the microbial rearing environment.

We have previously observed the microbiomes of RW and fish are highly dissimilar in aquaculture systems with cod larvae (Vestrum et al. 2020) and Atlantic salmon (Bugten et al. 2022), but that the similarity increased in systems where opportunistic bacteria were selected for in the RW. This increased similarity was due to higher abundances of a few opportunistic bacterial populations [one and two, respectively, in the studies of Bugten et al. (2022) and Vestrum et al. (2020)] both in the RW and the fish microbiomes. Thus, *r-*selection in the RW promoted the growth of a few bacterial populations that colonized the fish. This led to a lowered evenness of the fish microbiomes (Vestrum et al. 2020, Bugten et al. 2022). Based on the observations in these and the current study, we suggest that in *K-*selected systems, such as in most natural environments and some aquaculture systems (Vadstein et al. 2018), distinct bacterial populations are selected for in the water and on the mucosal surfaces of the fish. In such environments, the fish microbiomes are highly dissimilar from the environmental microbiomes. However, under conditions that favor the growth of opportunistic bacteria in the water, such as in systems with high organic loading and *r-*selection, some of these opportunistic bacteria will be able to colonize the fish, and thus be highly abundant in both the RW and the fish microbiomes. This leads to higher similarity between water and fish microbiomes, a lower evenness of the fish microbiomes, and potentially to detrimental fish–bacteria interactions.

## Conclusions

We found no effect of the host strain on the composition of the early gut microbiome of yolk sac fry of Atlantic salmon. We succeeded in creating *r-* and *K-*selected microbiomes in the water that was added to the rearing flasks upon the regular water exchanges, but *r-*selection acted on microbiomes in the RW of all flasks. Still, the composition of the RW microbiomes differed significantly between the flasks that had been added *r-* and *K-*selected water: In the Add-K flasks, they generally had lower alpha diversities and higher relative abundances of *Pseudomonas*. The gut microbiomes varied extensively between individuals, even within rearing flasks. Selection in the host structured the gut microbiomes, but stochastic processes explained the large interindividual variation. The microbiomes of the RW influenced the gut microbiomes: first, composition of gut microbiomes differed significantly between the Add-r and the Add-K flasks. Furthermore, in the Add-K flasks, the gut microbiomes were more similar in their community composition to the RW microbiomes, and the assembly of the gut microbiomes seemed to be less influenced by stochastic processes. A possible explanation is that in the RW of the Add-K flasks, there were higher relative abundances of a few opportunistic bacterial populations which were able to colonize the gut of the yolk sac fry. The fry in the Add-K flasks had lower growth rates than those in the Add-r flasks, probably a result of negative host–microbe interactions. The growth of the wild salmon strain appeared to be more influenced by the microbial rearing environment than the aquaculture strain. These findings highlight the importance of, but also the challenges related to, managing the microbial environment when cultivating fish.

## Supplementary Material

fiaf007_Supplemental_File

## References

[bib1] Andrews JH, Harris RF. R-selection and K-selection and microbial ecology. Adv Microbial Ecol. 1986;9:99–147.

[bib2] Antharam VC, Li EC, Ishmael A et al. Intestinal dysbiosis and depletion of butyrogenic bacteria in *Clostridium difficile* infection and nosocomial diarrhea. J Clin Microbiol. 2013;51:2884–92. 23804381 10.1128/JCM.00845-13 PMC3754663

[bib3] Attramadal KJK, Truong TMH, Bakke I et al. RAS and microbial maturation as tools for K-selection of microbial communities improve survival in cod larvae. Aquaculture. 2014;432:483–90.

[bib4] Bakke I, Coward E, Andersen T et al. Selection in the host structures the microbiota associated with developing cod larvae (*Gadus morhua*). Environ Microbiol. 2015;17:3914–24. 25923170 10.1111/1462-2920.12888

[bib5] Bledsoe JW, Peterson BC, Swanson KS et al. Ontogenetic characterization of the intestinal microbiota of channel catfish through 16S rRNA gene sequencing reveals insights on temporal shifts and the influence of environmental microbes. PLoS One. 2016;11:e0166379. 27846300 10.1371/journal.pone.0166379 PMC5113000

[bib6] Bledsoe JW, Waldbieser GC, Swanson KS et al. Comparison of channel catfish and blue catfish gut microbiota assemblages shows minimal effects of host genetics on microbial structure and inferred function. Front Microbiol. 2018;9:1073. 29875764 10.3389/fmicb.2018.01073 PMC5974930

[bib7] Bray JR, Curtis JT. An ordination of the upland forest communities of Southern Wisconsin. Ecol Monogr. 1957;27:326–49.

[bib8] Bugten AV, Attramadal KJK, Fossmark RO et al. Changes in rearing water microbiomes in RAS induced by membrane filtration alters the hindgut microbiomes of Atlantic salmon (*Salmo salar*) parr. Aquaculture. 2022;548:737661.

[bib9] Burns AR, Miller E, Agarwal M et al. Interhost dispersal alters microbiome assembly and can overwhelm host innate immunity in an experimental zebrafish model. Proc Nat Acad Sci USA. 2017;114:11181–6. 28973938 10.1073/pnas.1702511114 PMC5651736

[bib10] Burns AR, Stephens WZ, Stagaman K et al. Contribution of neutral processes to the assembly of gut microbial communities in the zebrafish over host development. ISME J. 2016;10:655–64. 26296066 10.1038/ismej.2015.142 PMC4817674

[bib11] Canny SGD, Nordgård CT, Mathisen AJH et al. A novel gnotobiotic experimental system for Atlantic salmon (*Salmo salar* L.) reveals a microbial influence on mucosal barrier function and adipose tissue accumulation during the yolk sac stage. Front Cell Infect Microbiol. 2023;12:1068302. 36817693 10.3389/fcimb.2022.1068302 PMC9929952

[bib49_114_161325] Clarke KR . Nonparametric multivariate analyses of changes in community structure. Aust J Ecol. 1993;18:117–43.

[bib12] Clerissi C, de Lorgeril J, Petton B et al. Microbiota composition and evenness predict survival rate of oysters confronted to Pacific Oyster Mortality Syndrome. Front Microbiol. 2020;11:311. 32174904 10.3389/fmicb.2020.00311 PMC7056673

[bib13] Cole JR, Wang Q, Fish JA et al. Ribosomal Database Project: data and tools for high throughput rRNA analysis. Nucleic Acids Res. 2014;42:D633–42. 24288368 10.1093/nar/gkt1244 PMC3965039

[bib14] De Schryver P, Vadstein O. Ecological theory as a foundation to control pathogenic invasion in aquaculture. ISME J. 2014;8:2360–8. 24892581 10.1038/ismej.2014.84 PMC4260705

[bib15] Dvergedal H, Sandve SR, Angell IL et al. Association of gut microbiota with metabolism in juvenile Atlantic salmon. Microbiome. 2020;8:160. 33198805 10.1186/s40168-020-00938-2 PMC7670802

[bib16] Edgar RC . UNOISE2: improved error-correction for Illumina 16S and ITS amplicon sequencing. bioRxiv. 2016a;081257.

[bib17] Edgar RC . SINTAX: a simple non-Bayesian taxonomy classifier for 16S and ITS sequences. bioRxiv. 2016b;074161.

[bib47_988_165725] Egerton S, Culloty S, Whooley J et al. The gut microbiota of marine fish. Front Microbiol. 2018;9:873. 29780377 10.3389/fmicb.2018.00873 PMC5946678

[bib18] Giatsis C, Sipkema D, Smidt H et al. The impact of rearing environment on the development of gut microbiota in tilapia larvae. Sci Rep. 2015;5:18206. 26658351 10.1038/srep18206 PMC4676014

[bib19] Hagerty SL, Hutchison KE, Lowry CA et al. An empirically derived method for measuring human gut microbiome alpha diversity: demonstrated utility in predicting health-related outcomes among a human clinical sample. PLoS One. 2020;15:e0229204. 32119675 10.1371/journal.pone.0229204 PMC7051054

[bib20] Hammer Ø, Harper DAT, Ryan PD. PAST: paleontological statistics software package for education and data analysis. Palaeontol Electron. 2001;4:9.

[bib21] Hill MO . Diversity and evenness: a unifying notation and its consequences. Ecology. 1973;54:427–32.

[bib22] Hillebrand H, Bennett DM, Cadotte MW. Consequences of dominance: a review of evenness effects on local and regional ecosystem processes. Ecology. 2008;89:1510–20. 18589516 10.1890/07-1053.1

[bib23] Johnston IA, Kent MP, Boudinot P et al. Advancing fish breeding in aquaculture through genome functional annotation. Aquaculture. 2024;583:740589.

[bib24] Karlsson S, Moen T, Lien S et al. Generic genetic differences between farmed and wild Atlantic salmon identified from a 7 K SNP-chip. Mol Ecol Resour. 2011;11:247–53. 21429178 10.1111/j.1755-0998.2010.02959.x

[bib25] Kim PS, Shin NR, Lee JB et al. Host habitat is the major determinant of the gut microbiome of fish. Microbiome. 2021;9:166. 34332628 10.1186/s40168-021-01113-x PMC8325807

[bib26] Koss DR, Bromage NR. Influence of the timing of initial feeding on the survival and growth of hatchery-reared Atlantic salmon (*Salmo-Salar* L). Aquaculture. 1990;89:149–63.

[bib27] Li WH, Liu JM, Tan H et al. Genetic effects on the gut microbiota assemblages of hybrid fish from parents with different feeding habits. Front Microbiol. 2018;9:2972. 30564218 10.3389/fmicb.2018.02972 PMC6288232

[bib28] Mathisen AJH . Effect of host strain and microbial water quality on the colonization of salmon fry. Master Thesis, NTNU, 2019.

[bib29] Michl SC, Ratten JM, Beyer M et al. the malleable gut microbiome of juvenile rainbow trout (*Oncorhynchus mykiss*): diet-dependent shifts of bacterial community structures. PLoS One. 2017;12:e0177735. 28498878 10.1371/journal.pone.0177735 PMC5428975

[bib30] Nemergut DR, Schmidt SK, Fukami T et al. Patterns and processes of microbial community assembly. Microbiol Mol Biol Rev. 2013;77:342–56. 24006468 10.1128/MMBR.00051-12 PMC3811611

[bib31] Ringo E, Zhou Z, Vecino JLG et al. Effect of dietary components on the gut microbiota ofaquatic animals. A never-ending story?. Aquacult Nutr. 2016;22:219–82.

[bib32] Sahlmann C, Gu J, Kortner TM et al. Ontogeny of the digestive system of Atlantic Salmon (*Salmo salar* L.) and effects of soybean meal from start-feeding. PLoS One. 2015;10:e0124179. 25923375 10.1371/journal.pone.0124179 PMC4414279

[bib48_987_160825] Saltveit SJ, Brabrand A. Incubation, hatching and survival of eggs of Atlantic salmon (Salmo salar) in spawning redds influenced by groundwater. Limnologica. 2013;43:325–31.

[bib33] Skjermo J, Salvesen I, Oie G et al. Microbially matured water: a technique for selection of a non-opportunistic bacterial flora in water that may improve performance of marine larvae. Aquacult Int. 1997;5:13–28.

[bib34] Stegen JC, Lin XJ, Fredrickson JK et al. Estimating and mapping ecological processes influencing microbial community assembly. Front Microbiol. 2015;6:370. 25983725 10.3389/fmicb.2015.00370 PMC4416444

[bib35] Vadstein O, Attramadal KJK, Bakke I et al. K-selection as microbial community management strategy: a method for improved viability of larvae in aquaculture. Front Microbiol. 2018;9:2730. 30487782 10.3389/fmicb.2018.02730 PMC6246659

[bib36] Vellend M . Conceptual synthesis in community ecology. Q Rev Biol. 2010;85:183–206. 20565040 10.1086/652373

[bib37] Vestrum RI, Attramadal KJK, Vadstein O et al. Bacterial community assembly in Atlantic cod larvae (*Gadus morhua*): contributions of ecological processes and metacommunity structure. FEMS Microbiol Ecol. 2020;96:fiaa163. 32816010 10.1093/femsec/fiaa163 PMC7456331

[bib38] Vestrum RI, Attramadal KJK, Winge P et al. Rearing water treatment induces microbial selection influencing the microbiota and pathogen associated transcripts of cod (*Gadus morhua*) larvae. Front Microbiol. 2018;9:851. 29765364 10.3389/fmicb.2018.00851 PMC5938384

[bib39] Webb CO, Ackerly DD, McPeek MA et al. Phylogenies and community ecology. Annu Rev Ecol Syst. 2002;33:475–505.

[bib40] Yan QY, Li JJ, Yu YH et al. Environmental filtering decreases with fish development for the assembly of gut microbiota. Environ Microbiol. 2016;18:4739–54. 27130138 10.1111/1462-2920.13365

[bib41] Yan QY, van der Gast CJ, Yu YH. Bacterial community assembly and turnover within the intestines of developing zebrafish. PLoS One. 2012;7:e30603. 22276219 10.1371/journal.pone.0030603 PMC3261916

[bib42] Yi BQ, Dalpke AH. Revisiting the intrageneric structure of the genus *Pseudomonas* with complete whole genome sequence information: insights into diversity and pathogen-related genetic determinants. Infect Genet Evol. 2022;97:105183. 34920102 10.1016/j.meegid.2021.105183

[bib43] Youngblut ND, Reischer GH, Walters W et al. Host diet and evolutionary history explain different aspects of gut microbiome diversity among vertebrate clades. Nat Commun. 2019;10:2200. 31097702 10.1038/s41467-019-10191-3 PMC6522487

[bib44] Zhang WZ, Chen RR, Meng FF et al. Ecosystem functioning is linked to microbial evenness and community composition along depth gradient in a semiarid lake. Ecol Indic. 2021;132:108314.

[bib45] Zhang ZM, Li DP, Refaey MM et al. Host age affects the development of southern catfish gut bacterial community divergent from that in the food and rearing water. Front Microbiol. 2018;9:495. 29616008 10.3389/fmicb.2018.00495 PMC5869207

[bib46] Zhou JZ, Ning DL. Stochastic Community Assembly: does it matter in microbial ecology?. Microbiol Mol Biol Rev. 2017;81:e00002–17. 29021219 10.1128/MMBR.00002-17 PMC5706748

