# Supplementary material for: Community assembly of gut microbiomes in yolk sac fry of Atlantic salmon: host genetics, environmental microbiomes, and ecological processes
Source: FEMS Microbiol Ecol. 2025 Jan 17;101(2):fiaf007. doi: 10.1093/femsec/fiaf007 (PMC11797051; doi:10.1093/femsec/fiaf007)

**Supplementary material**

**Figure S1. Bacterial density of the added (Aw) and rearing water (Rw) analyzed with flow cytometry.** Points indicate the average density and error bars the standard deviation. Samples were collected on water exchanges carried out after the first exposure to bacteria on seven days post hatching (dph) until the end of the experiment (22 dph). For water added to rearing flasks upon water exchanges, one sample was collected per treatment. For the rearing water, one sample per replicate flask was collected, i.e., a total of 16 rearing water samples per sampling time.


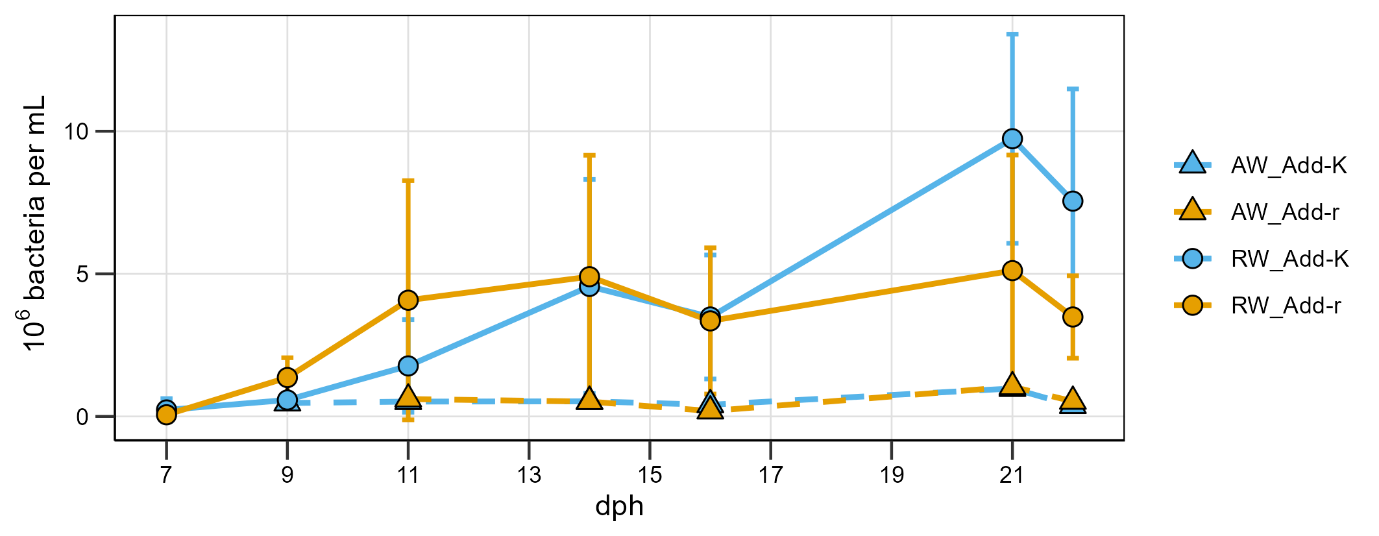


**Figure S2. Bray Curtis similarities for comparison of the gut microbiomes within and between Add-r and Add-K flasks.** For each rearing condition, there were four replicate rearing flasks, and individual guts were sampled from four individuals per flasks on 22 dph.


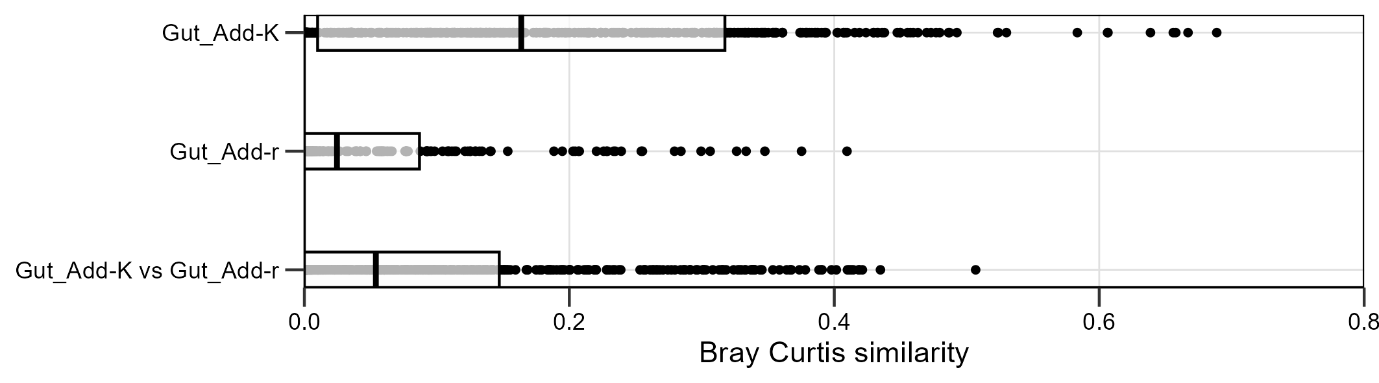


**Figure S3. The relative abundance of ten ASVs in gut samples that had been identified as the ten most abundant ASVs in the rearing water samples.** For each rearing condition, there were four replicate rearing flasks, and individual guts were sampled for four individuals per flasks. In the sample names, the first number refers to the rearing flask and the second the individual ID. Finally, r and K indicates that the flasks had been added *r-* and *K-*selected water, respectively (Add-r and Add-K flasks).


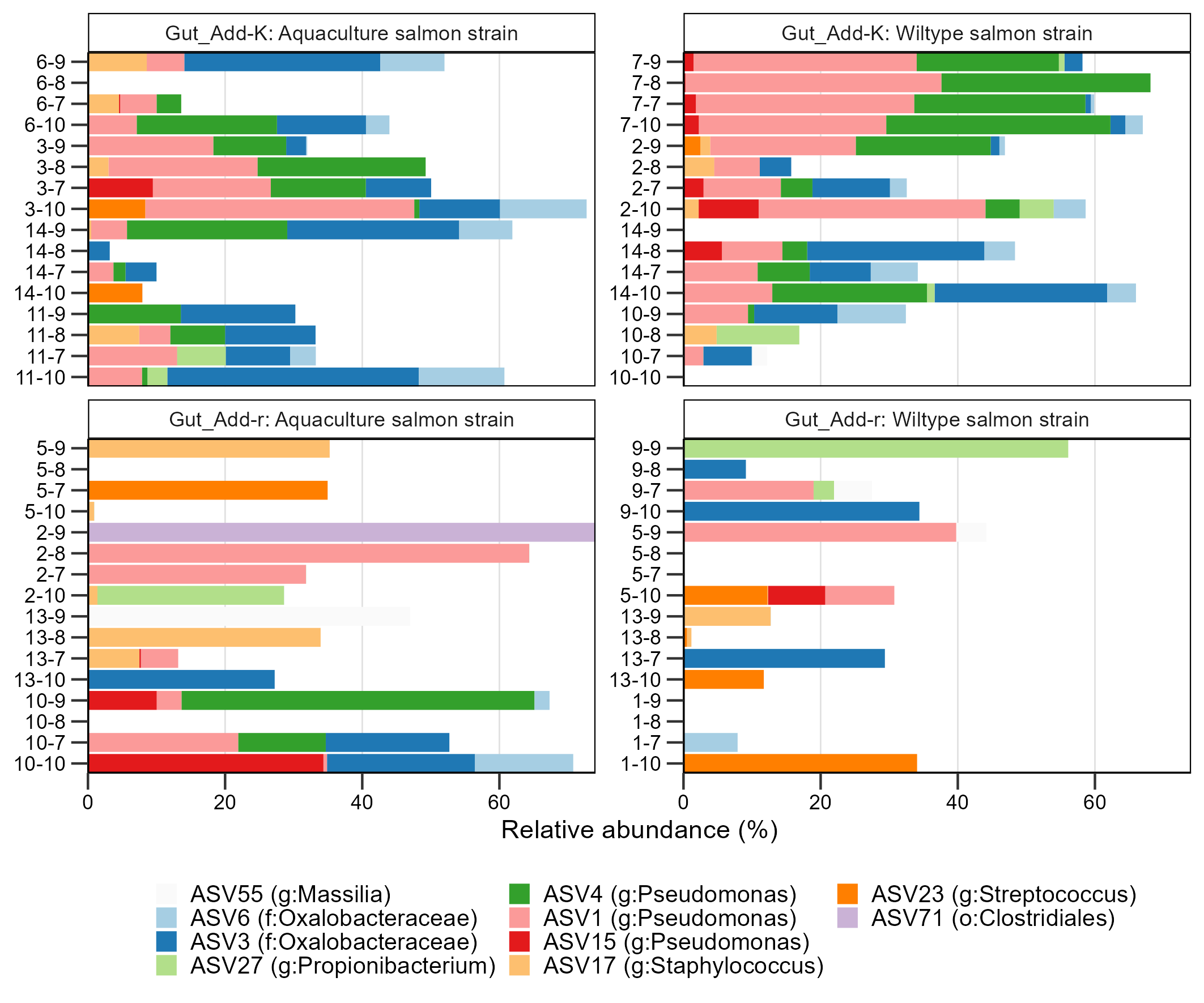


**Figure S4. Output from two-way ANOVA: body length on 22 dph of fish of the wild and aquaculture strain, reared in “Add-r” and “Add-K” flasks.** The two-way ANOVA was performed with fish strain (wild or aquaculture) and rearing water group (Add-r or Add-K flasks) as variables, and body length as response. For each of the four experimental groups, a total of 24 individuals were used (6 individuals from each of 4 replicate flasks). A) Test results, and B) Average lengths according to the variables.

A)

B) The mean of the body lengths (in mm) for fish. Wi: Wild salmon strain; Aq: Aquaculture salmon strain; K: fish reared in Add-K flasks, r: fish reared in Add-r flasks.


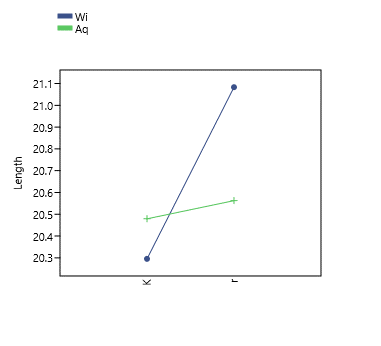

Supplement: fiaf007_Supplemental_File [file fiaf007_supplemental_file.docx]
